# Histopathological Findings of Steroid-Resistant Nephrotic Syndrome in Pediatric Patients at Queen Rania Children's Hospital: A Single-Center Experience

**DOI:** 10.7759/cureus.92311

**Published:** 2025-09-14

**Authors:** Mahdi Q Frehat, Aghadir Alhadidi, Moath Al Qawaqenah, Ruba S Al Assaf, Shawq Al Thaher, Amani Alrousan, Lubna Alkhatib, Mohammad F Sweiti, Batool Mansour

**Affiliations:** 1 Pediatric Nephrology, Jordanian Royal Medical Services, Amman, JOR; 2 Pediatrics, Jordanian Royal Medical Services, Amman, JOR; 3 Nephrology, Jordanian Royal Medical Services, Amman, JOR; 4 Pathology and Laboratory Medicine, Royal Medical Services-Princess Iman Center for Research and Laboratory, Amman, JOR; 5 Pediatrics and Child Health, Jordanian Royal Medical Services, Amman, JOR

**Keywords:** edema, histopathological diagnosis, minimal change disease, nephrotic syndrome, steroid-resistant nephrotic syndrome

## Abstract

Background: Steroid-resistant nephrotic syndrome (SRNS) is one of the most challenging conditions to be managed in pediatric nephrology that often requires a multi-step and individualized treatment approach; otherwise, it may continue to progress to end-stage renal disease. Histopathological evaluation with a kidney biopsy remains a cornerstone in the diagnosis, guiding the management through the identification of patterns such as focal segmental glomerulosclerosis and minimal change disease. Epidemiological data from the Middle East remain limited and underreported, with regional cohorts notably underrepresented in both genetic and therapeutic outcome studies. This study aims to characterize the histopathological findings of pediatric SRNS cases managed at Queen Rania Children’s Hospital, offering locally relevant insights to inform diagnostic and treatment strategies.

Method: This study is a retrospective study of medical records of pediatric patients with steroid-resistant nephrotic syndrome at Queen Rania Children’s Hospital in Jordan during the period from January 2020 to August 2024. It included demographics, early presentation, histopathological findings, and response to treatment.

Results: A total of 72 children were diagnosed with steroid-resistant nephrotic syndrome (SRNS), of whom 53% (n = 38) were female. The most frequent initial presentation was generalized edema. Minimal change disease (MCD) was the predominant histopathological finding, identified in 34.7% (n = 25) of cases across all age groups, while focal segmental glomerulosclerosis (FSGS) was more commonly observed in older children aged 8-14 years. A positive family history was reported in 18.1% (n = 13) of participants. Angiotensin-converting enzyme inhibitors (ACEIs) were the most frequently prescribed adjunct therapy with prednisolone, used in 51.4% (n = 37) of cases for both blood pressure management and antiproteinuric effect. Overall, 76.3% (n = 55) of the cohort exhibited a favorable prognosis, defined by sustained remission, stable renal function, and absence of progression to end-stage renal disease (ESRD) during the follow-up period. These outcomes were most commonly observed in patients with minimal change disease (MCD) and those who responded to adjunct therapy with ACE inhibitors, highlighting the potential impact of early intervention.

Conclusion: Pediatric SRNS requires individualized evaluation, as age-related diagnostic patterns and variable clinical presentations influence outcomes. While most patients responded favorably to targeted therapies, the risk of progression to ESRD still existed, as the underlying mechanisms of steroid resistance remain insufficiently understood. These findings support the need for personalized treatment approaches and ongoing research into the underlying immunologic, genetic, and histopathologic mechanisms driving steroid resistance.

## Introduction

Nephrotic syndrome is a common kidney disorder in children, characterized by significant proteinuria, hypoalbuminemia, hyperlipidemia, and generalized edema [1]. These symptoms not only affect the quality of life but also bring serious risks to long-term well-being and general health [1]. Corticosteroid therapy remains the first-line treatment for nephrotic syndrome. Its administration is highly effective, providing excellent therapeutic outcomes and leading to marked clinical improvement in most patients [2]. The response to such therapy is the most important prognostic factor influencing both immediate treatment decisions and long-term management strategies [3]. Among the various subtypes of nephrotic syndrome, steroid-resistant nephrotic syndrome (SRNS) is particularly concerning. It is defined by a failure to achieve complete remission despite adherence to the prescribed standard steroid regimen, which will carry a high risk of progression to end-stage renal disease (ESRD) [4]. In children, the standard treatment typically involves administering prednisolone at a dose of 60 mg/m² daily for four weeks, followed by 40 mg/m² every 48 hours for an additional four weeks [5,6].

The histopathologic lesions of the kidney in children with primary nephrotic syndrome have a bearing on the clinical course and the response to steroid therapy. More than 85% of children with primary nephrotic syndrome who undergo a kidney biopsy will have one of the idiopathic nephrotic syndrome patterns. Of these patients, 59% to 72% will respond completely to steroid therapy, while the remainder will either show no response or a poor response [7,8].

Treatment options for SRNS may include immunosuppressive agents, such as calcineurin inhibitors (e.g., tacrolimus) and non-biologic therapies like mycophenolate mofetil. Antihypertensive medications, particularly angiotensin-converting enzyme inhibitors (ACEIs), are frequently used to control blood pressure and for their antiproteinuric effects [9,10]. Effective management of SRNS is crucial in preventing progression to ESRD, highlighting the importance of ongoing research to improve therapeutic strategies and outcomes for pediatric patients affected by this challenging condition [3,9]. The aim of this study is to describe the histopathological patterns observed in pediatric patients diagnosed with steroid-resistant nephrotic syndrome (SRNS) at Queen Rania Children’s Hospital, and to assess their clinical relevance in guiding diagnosis and treatment decisions within a regional healthcare setting.

## Materials and methods

This retrospective observational study included 76 pediatric patients, aged 1 to 14 years, diagnosed with steroid-resistant nephrotic syndrome (SRNS), who underwent percutaneous renal biopsy at Queen Rania Children's Hospital (Amman, Jordan) between January 2020 and August 2024. Eligible participants were children aged 14 years or younger diagnosed with steroid-resistant nephrotic syndrome (SRNS), defined as persistent proteinuria following a minimum of four weeks of corticosteroid therapy. Inclusion required the availability of renal biopsy results, complete clinical and laboratory documentation, and a minimum follow-up duration of six months. Patients were excluded (a) if they had secondary causes of nephrotic syndrome, such as infection-associated presentations or drug-induced pathology, as renal biopsy was not indicated for such cases in our clinical protocol, (b) if they had received immunosuppressive therapy prior to admission, or (c) if they had incomplete records or concurrent systemic diseases that could confound renal assessment.

When comparing the histopathological patterns among different age groups, patients were categorized into two age-defined groups: Group I (1-7 years) and Group II (>7-14 years). Four cases were excluded due to insufficient data that precluded reliable assessment and inclusion. Data were retrieved from electronic medical records using the Hakeem system, the national health information platform implemented at Jordanian Royal Medical Services facilities. The sampling approach was non-probabilistic and purposive, based on the availability of complete cases within the study period.

Descriptive statistics were applied to summarize demographic and clinical variables. Categorical data were presented as frequencies and percentages, while continuous variables were reported as means ± standard deviation or medians with interquartile ranges, depending on distribution. Comparative analyses of categorical variables were performed using the Chi-square (χ²) test to evaluate associations between age groups and histopathological patterns. The alpha level of significance was set at 0.05. The margin of error was constrained to ≤5% at a 95% confidence interval.

## Results

Demographic data

A total of 72 children diagnosed with SRNS were included in the study; each underwent an ultrasound-guided percutaneous kidney biopsy as part of the diagnostic and evaluative process. Within the cohort, 53% (n = 38) of the participants were female, indicating a nearly balanced distribution between the two genders. Participants' age at first presentation ranged from 1 to 14 years; the calculated mean was 9.01 ± 3.9 years, indicating a diverse age distribution within the study population. Positive family history was found among 18.1% (n=13) of the cases, as shown in Table 1.

**Table 1:** Demographic distribution of patients with SRNS undergoing renal biopsy Gender and family history in pediatric steroid-resistant nephrotic syndrome (SRNS) cases.

	Cases (n^*^=72)	Prevalence %
Gender		
Female	38	53 %
Male	34	47 %
Family history		
NO	59	81.9%
YES	13	18.1%

Clinical results

The most common initial presentation among the cases was edema, observed in 50% (n=36) of the participants. This was followed by hematuria, which was present in 33.33% (n=24) of the cases. Other symptoms included malar rash, arthralgia, proteinuria, and various other types of rashes. In one patient, the diagnosis was made incidentally through abnormal kidney function test results; another one also presented with oliguria without the presence of any other symptoms, as shown in Table 2.

**Table 2:** Clinical features of the patients with SRNS Clinical features at the time of diagnosis among children with steroid-resistant nephrotic syndrome (SRNS), listed by frequency and prevalence.

Clinical feature	Cases (n^*^=72)	Prevalence %
Edema	36	50%
Haematuria	24	33.3%
SLE symptoms	5	6.9%
Protinourea	3	4.1%
Rash	2	2.7%
Oliguria	1	1.4%
Abnormal KFT	1	1.4%

Histopathological findings

The most frequently diagnosed condition within the study was minimal change disease (MCD), which accounted for 34.7% (n=25) of the cases. This was followed by focal segmental glomerulosclerosis (FSGS), which represented 16.66% (n=12) of the diagnoses. Other diagnoses that were observed but less frequently included systemic lupus erythematosus (SLE), immunoglobulin (Ig)A nephropathy, membranoproliferative glomerulonephritis (MPGN), post-streptococcal glomerulonephritis (PSGN), C3 glomerulonephritis, and rapidly progressive glomerulonephritis (RPGN), as mentioned in Table 3.

**Table 3:** Histopathological patterns of patients with SRNS Observed histological subtypes and their prevalence in children with steroid-resistant nephrotic syndrome (SRNS). MCD: Minimal change disease, FSGS: Focal segmental glomerulosclerosis, SLE: Systemic lupus erythematosus, IgA: Immunoglobulin A, MPGN: Membranoproliferative glomerulonephritis, PSGN: Post-streptococcal glomerulonephritis, RPGN: Rapidly progressive glomerulonephritis.

Histological pattern	Cases (n^*^=72)	Prevalence %
MCD	25	34.7 %
FSGS	12	16.6%
SLE	9	12.5%
IgA Nephropathies	9	12.5%
MPGN	7	9.7%
PSGN	5	6.9%
C3	3	4.1%
RPGN	1	1.4%
NORMAL	1	1.4%

Notably, MCD was slightly more common among children aged ≤7 years, comprising 52% of MCD cases (13 cases out of the total 25 cases of whom were diagnosed with MCD). Conversely, FSGS was more frequently identified in the >7 age group, accounting for 75% of its cases, with only 25% (3 cases out of the 12 cases of whom were diagnosed with FSGS) diagnosed in younger children. These patterns suggest possible age-related variations in disease manifestation, as illustrated in Table 4. However, this difference did not reach statistical significance (χ² = 2.41, p = 0.12).

**Table 4:** MCD and FSGS distribution by age in pediatric SRNS Distribution of minimal change disease (MCD) and focal segmental glomerulosclerosis (FSGS) diagnoses by age group in children with steroid-resistant nephrotic syndrome (SRNS), highlighting age-related prevalence trends.

Pattern	Age	Cases	Prevalence %
MCD (25)	<=7	13	52%
	>7	12	48%
FSGS (12)	<=7	3	25%
	>7	9	75%

Drugs used in the treatment of SRNS

In the treatment of steroid-resistant nephrotic syndrome (SRNS), the most commonly used drug alongside prednisolone was angiotensin-converting enzyme inhibitors (ACEI), prescribed in 51.4% (n=37) of cases for both blood pressure control and its antiproteinuric effect. Calcineurin inhibitors (CNIs), such as cyclosporine and tacrolimus, were the second most frequently used, accounting for 41.6% (n=30) of cases. Mycophenolate mofetil (MMF) was also commonly used, in 30.5% (n=22), as shown in Table 5.

**Table 5:** Adjunctive therapies in pediatric steroid-resistant nephrotic syndrome (SRNS) Frequency of supportive medications used, including calcineurin inhibitors (CNIs), mycophenolate mofetil (MMF), and anti-hypertensive medications.

Anti-hypertensive medications	Cases	% Cases
YES	37	51.4%
NO	35	48.6%
CNI’s		
Yes	30	41.6%
No	42	58.3%
Mycophenolate moftile		
Yes	22	30.5%
No	50	69.4%

Prognosis and outcome

The prognosis for steroid-resistant nephrotic syndrome (SRNS) varies significantly among patients. A favorable outcome is seen in 76.3% (n=55) of cases, with individuals showing improvement in their condition. Conversely, 15.2% (n=11) of patients may progress to end-stage renal disease (ESRD), requiring renal replacement therapy. Furthermore, a smaller group, accounting for 8.3% (n=6), has undergone kidney transplantation due to ongoing renal dysfunction.

## Discussion

This study provides a comprehensive overview of steroid-resistant nephrotic syndrome (SRNS) in a pediatric population, highlighting the demographic, clinical, and histopathological findings. The nearly balanced gender distribution (53% female and 47% male) aligns with existing literature, which suggests that SRNS does not have a strong gender predisposition, although slight variations have been noted in different populations [8,11,12]. A positive family history was documented in 18.05% of cases, supporting the hypothesis of a genetic predisposition in a subset of the population. This aligns with previous studies emphasizing the role of genetic mutations, particularly in early-onset SRNS, where hereditary factors such as NPHS2 or WT1 mutations have been identified [13]. Genetic testing in these cases could be beneficial in identifying familial or hereditary patterns, guiding treatment strategies, and enhancing long-term management and prognosis. Regarding clinical presentation, edema was the most commonly presented symptom, affecting 50% of participants, and this is consistent with its role as a major clinical sign of nephrotic syndrome. Hematuria was the second most frequent presentation (33.33%), a noteworthy finding given its lesser association with classic nephrotic presentations and its stronger correlation with glomerular inflammation. The presence of malar rash and arthralgia in some cases raises suspicion of systemic conditions, such as systemic lupus erythematosus (SLE), which has been documented to overlap with SRNS in pediatric patients. Notably, these patients had no prior diagnosis of such systemic conditions [9,14]. Histopathological analysis revealed a spectrum of diagnoses, including SLE, IgA nephropathy, membranoproliferative glomerulonephritis, post-streptococcal glomerulonephritis, C3 glomerulonephritis, and rapidly progressive glomerulonephritis. These results highlight the critical role of renal biopsy in identifying the underlying causes of SRNS, particularly in cases with non-specific clinical manifestations [5,9,14]. The study cohort’s age range (1-14 years, with a mean of 9.01 years) reflects the wide epidemiological span of SRNS in children. The age-dependent variability in clinical presentation and pathology is clinically significant. Children diagnosed in early childhood may exhibit genetically driven disease and poorer prognosis, whereas older children often present with secondary steroid resistance and distinct histological features [4,12]. Notably, focal segmental glomerulosclerosis (FSGS) was identified in 16.66% of cases and was more prevalent in patients over seven years of age. This supports previous data suggesting an association between age and FSGS occurrence and implies that older children may require more aggressive immunosuppressive therapy and rigorous follow-up due to worse long-term renal outcomes [15]. Interestingly, while previous studies have identified FSGS as the predominant histopathological subtype in idiopathic SRNS, excluding cases with hereditary forms, our data show minimal change disease (MCD) to be the most frequent diagnosis when both idiopathic and non-idiopathic forms are included [5,6,16]. This highlights the diagnostic diversity of SRNS and reinforces the need for a comprehensive diagnostic workup beyond clinical presentation alone. Overall, this study highlights the clinical and pathological heterogeneity of SRNS with respect to age, symptoms, and histopathology. The predominance of MCD among younger patients, while the highest proportion of FSGS cases was recorded among older age groups, suggests the need for tailored treatment strategies and supports further age-stratified research. These findings advocate the need for a multidisciplinary approach to improve the diagnostic precision and optimize therapeutic outcomes in affected children involving genetic testing, biopsy, and individualized immunosuppressive regimens [17].

While this study provides valuable insights into the clinical, histopathological, and therapeutic features of pediatric SRNS, several limitations may influence the interpretation of its findings. It was retrospective in nature and conducted at a single institution with a defined cohort of 72 patients, which may restrict the diversity of cases and limit broader applicability. However, Queen Rania Military Hospital is recognized as the largest pediatric referral center in Jordan and a leading facility in the region, and that enhances the relevance of these findings within similar clinical contexts. Also. The lack of genetic testing may limit the ability to explore monogenic forms of SRNS, which is important in understanding disease pathogenesis. Although histopathological assessments were carefully performed, minor inter-observer variability cannot be fully excluded. Additionally, long-term outcomes were not consistently available across all cases. These factors highlight the need for multicenter, prospective studies incorporating genetic, molecular, and longitudinal data to validate and expand upon these findings.

## Conclusions

Steroid-resistant nephrotic syndrome (SRNS) in children remains a diagnostic and therapeutic challenge that requires early recognition, individualized management, and long-term follow-up to prevent progression to chronic kidney disease. This study highlights the age-related histological patterns, common presenting symptoms, and management modalities that are currently being used. Further research is recommended for a better understanding of the underlying mechanisms of steroid resistance in pediatric SRNS. A deeper identification of the immunological, genetic, and histopathological patterns could guide more effective and individualized management strategies. Promoting this knowledge is essential to improving long-term outcomes and reduce disease burden.
